# Dose escalation study of intravenous and intra-arterial *N-*acetylcysteine for the prevention of oto- and nephrotoxicity of cisplatin with a contrast-induced nephropathy model in patients with renal insufficiency

**DOI:** 10.1186/s12987-017-0075-0

**Published:** 2017-10-03

**Authors:** Edit Dósa, Krisztina Heltai, Tamás Radovits, Gabriella Molnár, Judit Kapocsi, Béla Merkely, Rongwei Fu, Nancy D. Doolittle, Gerda B. Tóth, Zachary Urdang, Edward A. Neuwelt

**Affiliations:** 1 Heart and Vascular Center, Semmelweis University, 68 Városmajor Street, Budapest, 1122 Hungary; 2 1st Department of Internal Medicine, Semmelweis University, 26 Üllői Street, Budapest, 1085 Hungary; 3 Public Health & Preventive Medicine, Oregon Health & Science University, 3184 S.W. Sam Jackson Park Rd, CB669, Portland, OR 97329 USA; 4 Department of Neurology, Oregon Health & Science University, 3184 S.W. Sam Jackson Park Rd, L603, Portland, OR 97329 USA; 5 Department of Neurosurgery, Oregon Health & Science University, 3184 S.W. Sam Jackson Park Rd, L603, Portland, OR 97329 USA; 6 Portland Veterans Affairs Medical Center, 3710 S.W. US Veterans Hospital Rd, Portland, OR 97239 USA; 7 Blood–Brain Barrier and Neuro-Oncology Program, Oregon Health & Science University, 3181 S.W. Sam Jackson Park Road, L603, Portland, OR 97239 USA

**Keywords:** Ototoxicity, Nephrotoxicity, Chemoprotection, Clinical trial, Cisplatin, *N-*Acetylcysteine

## Abstract

**Background:**

Cisplatin neuro-, oto-, and nephrotoxicity are major problems in children with malignant tumors, including medulloblastoma, negatively impacting educational achievement, socioemotional development, and overall quality of life. The blood-labyrinth barrier is somewhat permeable to cisplatin, and sensory hair cells and cochlear supporting cells are highly sensitive to this toxic drug. Several chemoprotective agents such as *N-*acetylcysteine (NAC) were utilized experimentally to avoid these potentially serious and life-long side effects, although no clinical phase I trial was performed before. The purpose of this study was to establish the maximum tolerated dose (MTD) and pharmacokinetics of both intravenous (IV) and intra-arterial (IA) NAC in adults with chronic kidney disease to be used in further trials on oto- and nephroprotection in pediatric patients receiving platinum therapy.

**Methods:**

Due to ethical considerations in pediatric tumor patients, we used a clinical population of adults with non-neoplastic disease. Subjects with stage three or worse renal failure who had any endovascular procedure were enrolled in a prospective, non-randomized, single center trial to determine the MTD for NAC. We initially aimed to evaluate three patients each at 150, 300, 600, 900, and 1200 mg/kg NAC. The MTD was defined as one dose level below the dose producing grade 3 or 4 toxicity. Serum NAC levels were assessed before, 5 and 15 min post NAC. Twenty-eight subjects (15 men; mean age 72.2 ± 6.8 years) received NAC IV (N = 13) or IA (N = 15).

**Results:**

The first participant to experience grade 4 toxicity was at the 600 mg/kg IV dose, at which time the protocol was modified to add an additional dose level of 450 mg/kg NAC. Subsequently, no severe NAC-related toxicity arose and 450 mg/kg NAC was found to be the MTD in both IV and IA groups. Blood levels of NAC showed a linear dose response (p < 0.01). Five min after either IV or IA NAC MTD dose administration, serum NAC levels reached the 2–3 mM concentration which seemed to be nephroprotective in previous preclinical studies.

**Conclusions:**

In adults with kidney impairment, NAC can be safely given both IV and IA at a dose of 450 mg/kg. Additional studies are needed to confirm oto- and nephroprotective properties in the setting of cisplatin treatment.

*Clinical Trial Registration* URL: https://eudract.ema.europa.eu. Unique identifier: 2011-000887-92

## Background

Cisplatin is a common chemotherapeutic agent used to treat various types of malignant tumors. However, side effects such as neuro-, oto-, and nephrotoxicity limit the application of cisplatin. Cisplatin ototoxicity is of particular concern in children with malignant tumors where life-long hearing impairment can cause serious psychosocial deficits including social isolation, limited employment opportunities and associated earning potential, and an overall decrease in quality of life measures [1, 2]. The pathogenesis is not completely understood, but it is likely to be caused by depletion of intracellular glutathione (GSH) and generation of immune cell- and organ parenchymal-derived reactive oxygen species (ROS) and other free radicals [3]. Cisplatin is able to cross the blood-labyrinth barrier and enter the cochlea and sensory hair cells where it causes degeneration of the cochlear supporting cells, outer and inner hair cells and results in a progressive, irreversible hearing loss [4, 5]. For example, in medulloblastoma where the standard of care treatment includes cisplatin, ototoxicity occurs in approximately 80–90% of children treated with standard therapy [6]. Nephrotoxicity occurs in one-third of patients and can be potentially severe or life-threatening. Moreover, these toxicities utilize substantial healthcare resources and thus an inexpensive, effective, prophylactic protective strategy is of clear interest. Several oto- and nephroprotective approaches were developed (such as hydrating the patients during treatment, using less toxic cisplatin analogues) to avoid these reactions, including various chemoprotective agents used in experimental models (dimethylthiourea, melatonin, selenium, vitamin E, *N*-acetylcysteine [NAC], sodium thiosulfate) [5, 7–13].

*N*-Acetylcysteine is a sulfur-containing cysteine analog. It has been applied for decades as a mucolytic drug and as an antidote for acetaminophen overdose, as well as to prevent contrast-induced nephropathy (CIN) [14]. More recently, interest has been raised for the use of NAC in the prevention of cisplatin induced oto- and nephrotoxicity. Interestingly, CIN from iodine-based contrast agents and cisplatin share common mechanistic features including both intrinsic cellular- and inflammation-related ROS mediated cellular and stromal peroxidation damage [15–20]. The following properties of NAC are hypothesized to be paramount for the prevention of oto- and nephrotoxicity: (1) NAC is thought to act as a vasodilator through nitric oxide effects, thus improving blood flow, (2) NAC is a precursor to GSH, the body’s endogenous ROS scavenger, (3) the antioxidant properties of NAC dampen inflammation caused by damage-associated molecular patterns that arise from biological macromolecule peroxidation by ROS and cellular necrosis, and (4) NAC prohibits apoptosis and promotes cell survival by the activation of an extracellular signal-regulated kinase pathway [14]. When NAC enters the systemic circulation it can only leave the blood vessels after N-deacetylation and subsequent carrier mediated active transport of l-cysteine by the alanine–serine–cysteine transporter (ASC-1) [21]. Once in the brain, l-cysteine may act as an antioxidant or can be converted to GSH. Our group and others have shown a low level delivery of radiolabeled NAC across the BBB [22–24]. We demonstrated that even at very high NAC concentration (1200 mg/kg) delivery was less than 0.5% of the administered dose per gram tissue after intravenous (IV) administration in rats, but was significantly enhanced by intra-arterial (IA) administration [24]. It is possible that NAC is a ligand for ASC-1 prior to deacetylation or that NAC is rapidly deacetylated in the blood and the observed radioactivity in the brain was due to radioactive cysteine. In the setting of inflammation, oxidative stress could impair the BBB to increase NAC leak [22, 23]. In case of a brain tumor, vessels supplying the tumor possess impaired barrier properties so both NAC and cisplatin can enter the tumor tissue to some degree.

A literature review revealed 38 trials evaluating NAC in the prevention of CIN, 15 with positive and 23 with negative outcomes, and 17 meta-analyses with conflicting conclusions [25]. There has been significant heterogeneity between studies due to various routes of administration and different dosages [25, 26]. Most trials followed Tepel’s regimen of 600 mg of NAC orally twice a day for 48 h and 0.45% saline intravenously, before and after injection of the contrast agent, or placebo and saline as control [26].

Similarly to CIN, NAC has demonstrated mixed results in the literature as an otoprotectant in the context of cisplatin therapy [7, 14, 27–30]. Still, a handful of reports with positive results suggest otoprotective properties during cochlear insults through ROS mediated mechanisms. Dosing, route, and timing of NAC administration seem to be important variables in NAC medicated otoprotection. Whether or not NAC trafficking into the extravascular cochlear compartment occurs is an understudied question, and hence extravascular trafficking may not be required for otoprotective activity. NAC could potentially act by intravascular activity on ROS producing immune cells which can compromise blood-labyrinth barrier integrity and thus prevent enhanced cochlear uptake of cisplatin.

Preclinical ototoxicity studies demonstrated that IV or IA administration of NAC is required to achieve high blood concentration necessary for otoprotection [7, 8, 28, 31]. As Stenstrom observed, oral NAC is cleared via the portal vein on the first pass through the liver, however 31 of 38 reviewed trials ignored this first pass clearance and gave very small doses [14]. We assume that either the oral route or the applied low IV doses were likely a large factor in the negative results seen in previous clinical trials. We hypothesized that NAC at high IV and IA (via the descending aorta) doses can be injected with an acceptable toxicity profile in children with malignant tumors. Our primary goal was to perform a dose escalation study in pediatric patients. Due to rejection of our pediatric toxicity trial by the Institutional Review Board this phase I study used an adult population of subjects with stage 3 or worse kidney failure undergoing a radiologic procedure requiring iodine-based contrast media. Patients with renal failure were chosen with the thought that this population would be particularly sensitive to adverse events and thus the observed maximum tolerated dose (MTD) would include a large margin of safety when translated to the pediatric population. Using this study design we were also able to not only examine the chemoprotective properties of NAC, but could confirm its protection against CIN. The MTD will be evaluated for efficacy in a future trial, specifically in pediatric populations.

## Methods

### Study protocol

This was a prospective, non-randomized, single center dose escalation trial of patients with stage 3 or worse chronic renal disease (glomerular filtration rate [GFR] < 60 mL/min/1.73 m^2^) who underwent a digital subtraction angiography (DSA) and/or vascular intervention with an isotonic nonionic contrast material (Iodixanol) between the years 2012 and 2015. Indication for the procedures was established by a vascular team including vascular surgeons, interventional radiologists, and vascular physicians. Interventions were carried out according to international guidelines. Our primary objective was to establish the MTD of both IV and IA NAC. The secondary objective was to determine NAC pharmacology given IV or IA.

The study was approved by the Institutional Review Committee (12935-0/2011-EKL) and all subjects gave written informed consent.

#### Eligibility requirements

Patients between 18 and 85 years of age at risk for CIN were eligible to participate if they had stage 3 or worse kidney impairment (renal failure staging was determined by the following formula: Modification of Diet in Renal Disease, GFR ([mL/min/1.73 m^2^] = 175 × [Serum creatinine]^−1.154^ × [Age]^−0.203^ × [0.742 if the subject was female]) with a life expectancy of 4 weeks from the date of registration [32].

#### Exclusion criteria

Subjects were excluded if they had acute kidney injury (e.g., significant change over 4 weeks), were on dialysis, had a systolic blood pressure of < 90 mmHg, had decompensated heart failure at the time of admission, had a history of severe reactive airway disease, were at high risk for general anesthesia, were pregnant, had a positive serum human chorionic gonadotropin or was lactating, or who had contraindications to NAC or the contrast agent.

### Treatment plan

#### Dose escalation

**Fig. 1:**
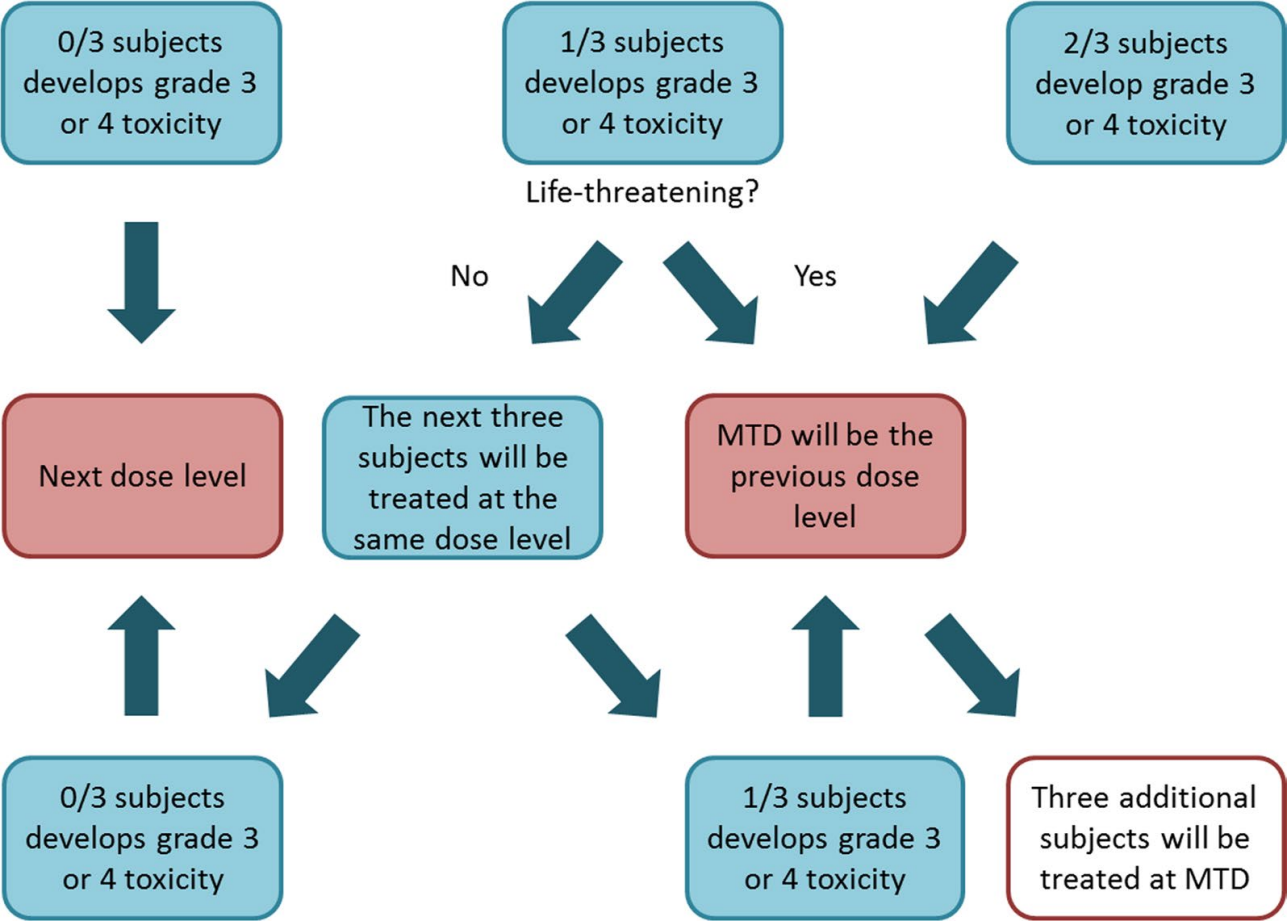
Dosing algorithm for *N*-acetylcysteine to determine the maximum tolerated dose in adults with chronic kidney disease. The *N*-acetylcysteine maximum tolerated dose was defined as one dose level below the dose that produced grade 3 or 4 toxicity. *MTD* maximum tolerated dose

A group of three subjects was aimed to be evaluated at each of the following fixed dose levels of NAC: dose level 1, 150 mg/kg/day; dose level 2, 300 mg/kg/day; dose level 3, 600 mg/kg/day; dose level 4, 900 mg/kg/day; and dose level 5, 1200 mg/kg/day. The first dose level was based on the standard of care treatment of acetaminophen overdose [33]. The dose escalation was evaluated by the rate of grade 3 or 4 toxicities. In case of a severe toxicity reaction an additional dose level was added. Toxicity was graded according to National Cancer Institute Common Terminology Criteria for Adverse Events version 3.0. [34]. The dosing algorithm can be seen in Fig. 1. The NAC MTD was defined as one dose level below the dose that produced grade 3 or 4 toxicity.

#### Assignment for IV versus IA NAC

Patients were assigned to IV or IA using the last digit of their hospital identification number. Those with even last digits received IV NAC and those with odd last digits received IA NAC. If the MTD was achieved for one group, all subsequent subjects were treated with the other regimen.

#### Premedication

Since it has been previously shown that NAC has dose dependent anaphylactoid reactions in 23–48% of patients, all participants received premedication prior to NAC administration [35]. Premedication regimen consisted of 100 mg IV methylprednisolone and 50 mg ranitidine 3 h prior to NAC, and 50 mg diphenhydramine 10 min prior to NAC. Additional doses of 25 mg diphenhydramine were given 10 min after the start of the NAC infusion and repeated as clinically indicated.

#### Administration of the study drug

The study drug (Acetylcysteine [Fluimucil Antidote]) is available as a 20% solution in 25 mL (200 mg/mL) single dose glass vials. The NAC was diluted to 150, 300, 450, and 600 mg/kg in 250 mL of diluent (5% dextrose in water [Isodex]). The 900 and 1200 mg/kg doses were designed to be diluted in 500 mL of diluent. Each of the above dilutions were given either IV through a peripheral vein using an infusion pump (Alaris GH) or IA down the descending aorta through a fluid injection system (Medrad Avanta) over a period of 25–55 min. In the case of IV injection, the flow rate was 1000 mL/h. For IA administration a pigtail catheter was used (the tip of the catheter was positioned at the level of the renal arteries) and a pulsed infusion of 16.5 mL volume at 16.5 mL/sec was performed and repeated for a total of 15 injections. In the event of grade 1 or 2 toxicities the infusion rate was reduced.

#### Subject monitoring

Vital signs (pulse and respiration rate), blood pressure, electrical activity of the heart, and oxygen saturation were recorded by a cardiologist at baseline, prior to NAC infusion, every 10 min during infusion, and for 30 min after completion of NAC infusion. The patient was closely monitored for anaphylactoid reaction throughout the endovascular procedure in the angiogram suite and in the recovery unit after the DSA and/or intervention. In the recovery unit, fluid intake and output were measured for 2–4 h until the subject was sent to the ward.

### Laboratory analysis of the blood samples

Blood samples were taken at baseline, prior to NAC, then 5 and 15 min after the NAC administration, as well as 24, 48, and 72 h following the radiologic procedure. Study drug and GSH levels were assessed prior to, then 5 and 15 min after the completion of the study drug infusion. Serum NAC and GSH analyses were done in our Research Laboratory using a high-performance liquid chromatography assay. Details of this procedure have been described previously [28].

### Statistical analysis

Statistical analysis was performed with SPSS 21.0 software (IBM Corp., Armonk, NY) and SAS 9.4 software (SAS Institute Inc., Cary, NC). Continuous variables were expressed as means and standard deviations and were compared between two groups using the Students’ *t* test. A linear mixed-effects model was applied to evaluate dose response relationships and differences at various time points for pharmacological factors while accounting for correlations among the multiple observations within the same patient. All analyses were two-tailed, and values of p ≤ 0.05 were considered statistically significant.

## Results

### Patient data

Twenty-eight subjects (13 women, 15 men; mean age: 72.2 ± 6.8 years) were enrolled. Fifteen subjects had DSA (lower or upper extremity angiography, N = 6; aortic arch and selective four-vessel cerebral angiography, N = 3; lower extremity plus aortic arch and selective four-vessel cerebral angiography, N = 3; renal angiography, N = 3) while 12 underwent percutaneous transluminal angioplasty with or without stent placement (internal carotid artery stenting, N = 4; renal artery stenting, N = 2; crural artery percutaneous transluminal angioplasty, N = 2; subclavian artery stenting, N = 1; common iliac artery stenting, N = 1; common iliac artery plus renal artery stenting, N = 1; superficial femoral artery stenting, N = 1). One patient (7_IV) did not have radiologic intervention due to NAC-related acute severe toxicity.

### *N*-Acetylcysteine toxicity

The administered NAC volume and NAC infusion time did not differ significantly between the corresponding IV and IA groups (Table 1).

**Table 1:** *N*-Acetylcysteine and contrast agent volumes, *N*-acetylcysteine administration time, baseline serum creatinine levels, and 5-min *N*-acetylcysteine and glutathione concentrations

Parameter	NAC dose (mg/kg)	IV group (Mean ± SD)	IA group (Mean ± SD)	p value
NAC volume (mL)	150	63.25 ± 50.61	51.88 ± 6.6	0.31
	300	109.5 ± 6.89	132.5 ± 46.37	0.183
	600	294	NA	NA
	450	167.58 ± 44.63	166.13 ± 41.61	0.956
NAC administration time (min)	150	26.67 ± 2.89	31.67 ± 7.53	0.196
	300	31.67 ± 5.77	38.33 ± 2.89	0.173
	600	51	NA	NA
	450	40 ± 6.32	42.5 ± 8.22	0.569
CA volume (mL)	150	94.33 ± 69.89	109.17 ± 56.16	0.768
	300	80 ± 39.69	86.67 ± 64.29	0.887
	600	0	NA	NA
	450	89.5 ± 15.86	88.5 ± 25.81	0.937
Baseline serum creatinine (μmol/L)	150	201 ± 11.97	118.17 ± 15.53	0.402
	300	123.67 ± 33.23	160 ± 24.76	0.343
	600	157	NA	NA
	450	209.33 ± 54.4	171.5 ± 52.89	0.486
NAC concentration at 5 min (mM)	150	0.43 ± 0.1	1.66 ± 0.32	*< 0.001*
	300	1.04 ± 0.6	3.12 ± 0.77	*0.023*
	600	4.53	NA	NA
	450	2.03 ± 0.95	4.1 ± 1.22	*0.009*
GSH concentration at 5 min (mM)	150	0.13 ± 0.16	0.2 ± 0.04	0.514
	300	0.13 ± 0.03	0.96 ± 0.36	0.058
	600	0.21	NA	NA
	450	0.19 ± 0.04	0.89 ± 0.31	*0.003*

Italicized p-values indicate statistically significant values

*IV* intravenous, *IA* intra-arterial, *SD* standard deviation, *NAC N*-acetylcysteine, *NA* not applicable, *CA* contrast agent, *GSH* glutathione

#### Maximum tolerated IV dose

**Fig. 2:**
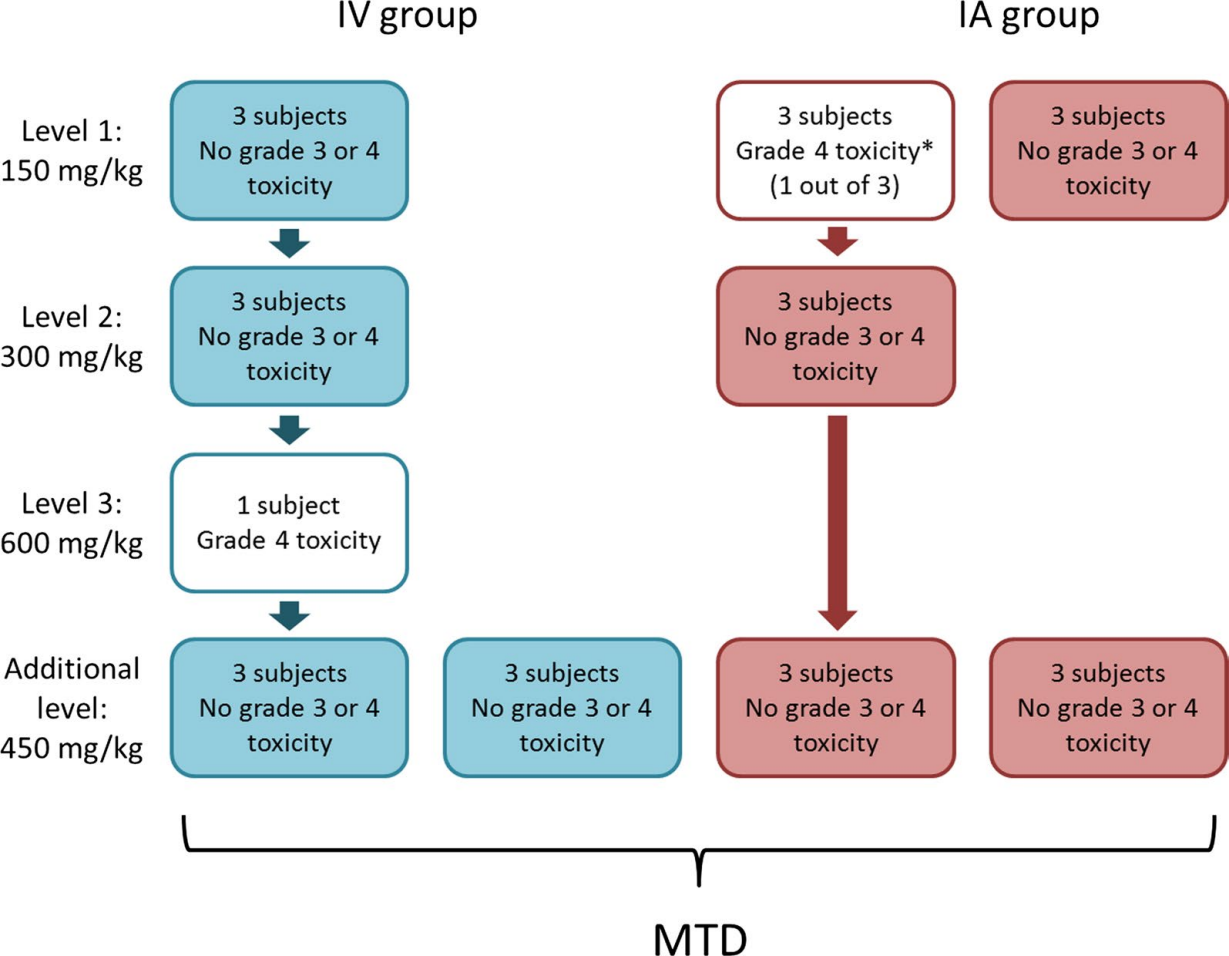
Establishment of the maximum tolerated dose for *N*-acetylcysteine in adults with chronic kidney disease. Four hundred and fifty mg/kg *N*-acetylcysteine was found to be the maximum tolerated dose in both intravenous and intra-arterial groups. Asterisk: Adverse reaction to contrast agent rather than to *N*-acetylcysteine. *IV* intravenous, *IA* intra-arterial, *MTD* maximum tolerated dose

Thirteen participants received IV NAC. Three patients completed dose level 1 and three completed dose level 2 without having grade 3 or 4 toxicity. The first subject (7_IV) enrolled to dose level 3 developed rashes, flushing, pruritus, and an intense bronchospasm immediately after completion of the study drug administration which rapidly progressed to respiratory and cardiac arrest. Successful cardiorespiratory resuscitation was performed according to the 2010 American Heart Association guidelines at which point the participant was transported to the intensive care unit where he was monitored for 3 days [36]. The patient left the hospital 6 days post NAC in good condition. Due to the serious toxicity in this subject, the protocol was modified and a new dose level of 450 mg/kg NAC was inserted between the 300 and 600 mg/kg doses. Participants 8_IV, 9_IV, and 10_IV received 450 mg/kg dose of NAC. None had grade 3 or 4 toxicity, therefore 450 mg/kg was considered to be the MTD and three additional patients were treated with the same dose in order to gain more data on NAC toxicity and pharmacokinetics (Fig. 2).

#### Maximum tolerated IA dose

Fifteen subjects received IA NAC. The first participant (1_IA) enrolled to the group developed an anaphylactic reaction with life-threatening symptoms. She was treated according to the 2011 World Allergy Organization anaphylaxis guidelines in the angiogram suite and was transported to the intensive care unit where she was monitored for 3 days [37]. The patient left the hospital 5 days post NAC infusion in good condition. The anaphylactic reaction occurred immediately after completion of the DSA. The time interval between the anaphylactic reaction and NAC infusion was 1 h. The case was discussed by a multidisciplinary team which considered the adverse reaction to be a consequence of the contrast agent rather than NAC based on the elapsed time from the study drug infusion to the time of the anaphylactic reaction. Also, the subject provided information after the adverse reaction that she developed hives on her chest 2 months previously after a cardiac catheterization. Furthermore, the cardiac catheterization was done in a different hospital, the hives were not mentioned in the final report, and the participant answered no for the question whether she had allergic reaction to anything in her life both prior to study enrollment and before the interventional procedure. Although two additional participants completed dose level 1 without having grade 3 or 4 toxicity, three more patients were treated with the same dose. Neither 300 nor 450 mg/kg dose produced severe toxicity. The 450 mg/kg dose was considered to be the MTD and three additional subjects received that dose (Fig. 2).

#### Minor toxicities

Grade 1 or 2 toxicities were seen in six participants (21.4%). Two-thirds of the minor toxicities occurred at a dose of 450 mg/kg NAC. All of them resolved either spontaneously or by giving appropriate treatment over 30 min to 12 h following the toxicity (Table 2).

**Table 2:** Toxicities attributed to *N*-acetylcysteine

Group	Patient number	Weight (kg)	NAC dose (mg/kg)	NAC volume (mL)	NAC toxicity
IV	4_IV	84	300	126	Grade 1, facial erythema
	7_IV	98	600	294	Grade 4, respiratory and cardiac arrest
	10_IV	96	450	216	Grade 2, pruritus and rash
	11_IV	103	450	231.75	Grade 1, coughing
IA	1_IA	55	150	41.25	Grade 4^a^, anaphylaxis
	8_IA	101	300	151.5	Grade 1, nausea
	10_IA	95	450	213.75	Grade 1, coughing
	11_IA	80	450	180	Grade 1, facial erythema

*NAC N*-acetylcysteine, *IV* intravenous, *IA* intra-arterial

^a^Adverse reaction to contrast agent rather than to NAC

### *N*-Acetylcysteine pharmacokinetics

**Fig. 3:**
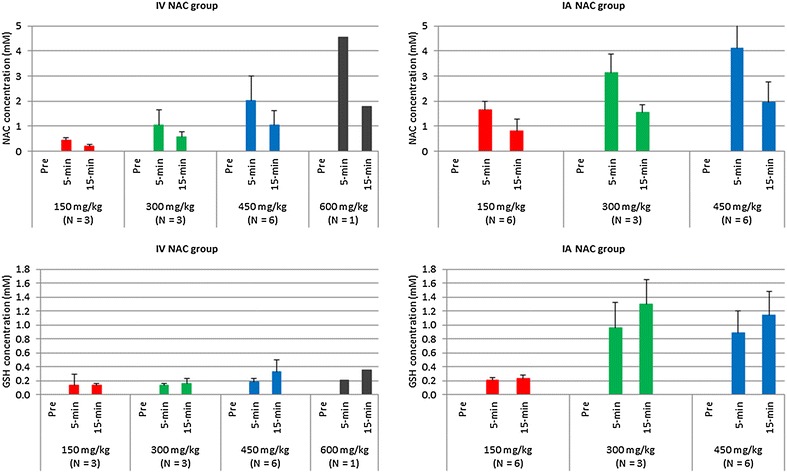
*N*-Acetylcysteine pharmacokinetics: serum concentrations of *N*-acetylcysteine and glutathione at different dose levels and time intervals. Blood levels of *N*-acetylcysteine (upper row) showed a significant linear dose response, while gluthatione concentrations (lower row) were inconsistently elevated. *IV* intravenous, *IA* intra-arterial, *NAC N*-acetylcysteine, *GSH* glutathione

Results of the high-performance liquid chromatography analysis are summarized in Fig. 3.

#### Serum NAC levels

At baseline, NAC and GSH were not detected in the serum samples. Blood levels of NAC showed a significant linear dose response both 5 min (slope, 1.07 mM increase for every 150 mg/kg rise in NAC dose; p = 0.001) and 15 min after IV administration (slope, 0.48 mM increase for every 150 mg/kg rise in NAC dose; p = 0.002). Similar significant linear dose responses were observed after IA injection with a slope of 1.22 mM for every 150 mg/kg increase in NAC dose at 5 min (p < 0.001) and with a slope of 0.58 mM for every 150 mg/kg increase in NAC dose at 15 min (p = 0.005). In each group, NAC levels were nearly halved from 5 to 15 min post infusion. In particular, the overall mean NAC level was 1.63 mM (SE: 0.23) and 0.81 mM (SE: 0.21), respectively, 5 and 15 min after IV administration; and 2.93 mM (SE: 0.22) and 1.42 mM (SE: 0.15), respectively, 5 and 15 min after IA injection. At 5 min post infusion, NAC concentrations were significantly higher in the IA groups compared to the corresponding IV groups (p < 0.001; p = 0.023, p =  0.009, respectively) (Table 1, Fig. 3).

#### Serum GSH levels

A significant linear dose response relationship was noted for GSH concentrations in the IV group. Since the relationships were similar at 5 and 15 min, an overall dose response relationship was estimated to yield a 0.37 mM increase in GSH values for every 150 mg/kg rise in NAC dose (p < 0.001). In contrast to patients in the IV group, the overall dose response relationship was not significantly linear in the IA group (p = 0.068). The mean GSH concentrations were higher in the IA than in the IV group (Table 1, Fig. 3).

## Discussion

Our goal was to provide the MTD of NAC as a correct scientific basis for future efficacy trials, particularly in pediatric populations. Four hundred and fifty mg/kg NAC was found to be the MTD in this study, and we have shown that it can be given with an acceptable toxicity both IV and IA in adults with impaired kidney function undergoing DSA with or without intervention. By determining the MTD we potentially gain the maximum concentration of NAC in the brain and cochlea to diminish the toxicity of agents like cisplatin, although the entry of NAC may be limited by the BBB and blood-labyrinth barrier.

A key factor in previous failed trials with NAC is that oral NAC is known to have 5–10% bioavailability in humans due to extensive first pass metabolism to GSH [38]. Oral NAC reaches a serum peak about an hour after ingestion and has an elimination half-life of 2.27 h [39]. Furthermore, there is no clear evidence that NAC effects are mediated indirectly by its metabolites.

The potential of oral NAC to be oto- and nephroprotective was examined in several preclinical studies. Dickey et al. determined in rats, that a single IV administration of 1500 mg/kg NAC is non-toxic, and three IV injections of 1200 mg/kg NAC, 4 h apart, are safe and well-tolerated [28]. In another study by Dickey et al. rats received NAC infusion at 100, 400 and 1200 mg/kg IV. Blood samples were taken 15 min post inoculation. Another group of rats was given NAC 1200 mg/kg orally, with blood samples collected after 15 and 60 min. Total NAC concentrations were analyzed and similarly to our findings, blood levels of NAC showed a rough linear dose response after IV administration of NAC. In contrast to the IV results, the group given NAC 1200 mg/kg by the oral route had very low levels of serum NAC [28]. In their third study, rats were treated with cisplatin 10 mg/kg intraperitoneally 30 min after NAC 400 mg/kg given by intraperitoneal, oral, or IV routes, compared with cisplatin alone. NAC was chemoprotective against the cisplatin nephrotoxicity, depending on the route of administration. Rats receiving NAC IV had very low blood urea nitrogen levels 3 days post treatment. In the case of oral or intraperitoneal NAC administration, the blood urea nitrogen concentrations were as high as in the group of rats who did not get NAC [28]. In their fourth study, rats were treated with cisplatin 10 mg/kg intraperitoneally 30 min after NAC 50 mg/kg infused IV or IA. The blood urea nitrogen levels were significantly lower in the IA group—the blood urea nitrogen levels were similar to those when NAC was injected IV at high dose (400 mg/kg)—indicating a significantly reduced rate of nephrotoxicity for the IA delivery [28]. Assuming that this rat chemoprotective model represents the effects of cisplatin as those of contrast agents in humans, these observations call into question if oral NAC or low dose IV NAC has any clinical impact on cisplatin induced oto- and nephrotoxicity.

Briguori et al. and Marenzi et al. were the only investigators who made dose comparisons in humans. Briguori et al. compared single dose NAC 600 mg orally twice a day for 48 h with double dose NAC 1200 mg (17.1 mg/kg for a 70 kg subject) orally twice a day for 48 h. Although these doses were not high and were given orally, the outcome was favorable for the double dose [40]. Marenzi et al. compared two IV doses (600 and 1200 mg total dose per patient) prior to the angioplasty and two oral doses (600 and 1200 mg twice a day for 48 h) after the procedure with placebo. A greater increase in serum creatinine was observed in the placebo group compared to patients treated with NAC and the higher NAC IV dose was even better than the lower dose, which implies that NAC actions may be dose dependent [41]. These observations are in line with the findings of the above mentioned rat studies and demonstrate the importance of this phase I trial.

It is also worth considering that the route of NAC administration markedly affects its biodistribution. In an animal study performed by our group, we found that when radiolabeled NAC was administered IA into the right carotid artery of the rat, high levels of radiolabel were found throughout the right cerebral hemisphere, regardless of whether or not the BBB was opened. Delivery was 0.41% of the injected dose, comparable to the levels found in the liver (0.57%) and kidney (0.70%). In contrast, the aortic infusion above the renal arteries prevented the brain delivery and changed the biodistribution of NAC. The change in tissue delivery with different modes of administration is likely due to NAC being deacetylated and the amino acid cysteine is rapidly bound by tissues via the amino acid transporters [24].

The limitations of our study include the special subject population: all patients were older than 50 years, had impaired kidney function, and atherosclerotic disease. Although serum creatinine values were measured both before and after contrast agent administration additional trials should be performed to determine whether either IV or IA 450 mg/kg NAC is protective against CIN or chemoprotective against cisplatin in pediatric subjects.

## Conclusions

In conclusion, we found that NAC can be safely given both IV and IA at a dose of 450 mg/kg in adults with reduced renal function. Phase II and III studies are needed to determine whether high IV and IA doses can avoid oto- and nephrotoxicity of platinum-based chemotherapy, and if yes, whether a particular route of administration of NAC provides improved chemoprotection. A considerable hurdle with NAC is disentangling the mixed results from studies utilizing oral NAC administration; we advocate for careful analysis and comparison of oral route trials in humans with those of IV or IA. A phase I trial in children is currently underway with different doses of NAC after cisplatin to prevent ototoxicity (clinicaltrials.gov NCT02094625).

## Abbreviations

ASC-1: alanine–serine–cysteine transporter
BBB: blood–brain barrier
CIN: contrast-induced nephropathy
DSA: digital subtraction angiography
GFR: glomerular filtration rate
GSH: glutathione
IA: intra-arterial
IV: intravenous
MTD: maximum tolerated dose
NAC: *N*-Acetylcysteine
ROS: reactive oxygen species
